# Supplementary material for: Early Locus Coeruleus noradrenergic axon loss drives olfactory dysfunction in Alzheimer’s disease
Source: Nat Commun. 2025 Aug 8;16:7338. doi: 10.1038/s41467-025-62500-8 (PMC12334674; doi:10.1038/s41467-025-62500-8)
Supplement: Supplementary file 1 — Supplementary Information [file 41467_2025_62500_MOESM1_ESM.pdf]

Supplementary figures

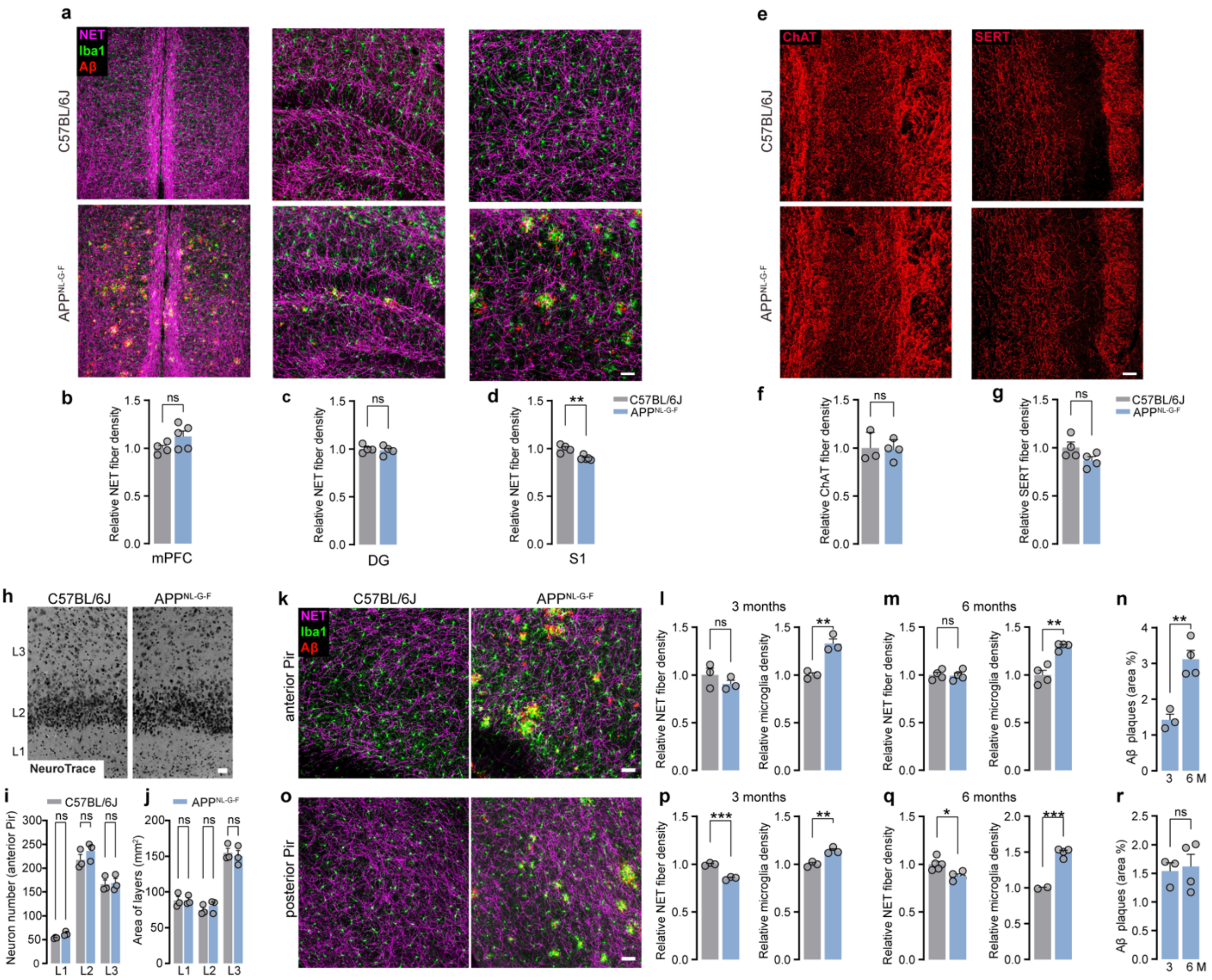

Supplementary Fig. 1: Axon density in different brain regions

a, Immunostaining against LC axons (NET, magenta), microglia (Iba1, green) and Aβ plaques (Aβ, red) in the medial prefrontal cortex (mPFC), dentate gyrus of the hippocampus (DG) and in the somatosensory cortex (S1) of 6 months old C57BL/6J and $APP^{NL\text{-}G\text{-}F}$ mice. Scale bar: 50 µm. **b**, Quantification of LC axon density in the mPFC. **c**, Quantification of LC axon density in the DG. **d**, Quantification of LC axon density in in the S1 cortex. **e**, Staining of cholinergic (ChAT, red) and serotonergic (SERT, red) axons in the olfactory bulb of 3 months old C57BL/6J and $APP^{NL\text{-}G\text{-}F}$ mice. **f**, Quantification of cholinergic axon density. **g**, Quantification of serotonergic density. **h**, NeuroTrace stain visualizing neurons in the different layers of the anterior piriform cortex (Pir). Scale bar: 50 µm. **i**, Quantification of neuron number per layer, comparing C57BL/6 animals to $APP^{NL\text{-}G\text{-}F}$ animals. **j**, Quantification of area per layer. **k**,

Immunostaining against LC axons (NET, magenta), microglia (Iba1, green) and Aβ plaques (Aβ, red) in the anterior piriform cortex. Scale bar: 50 µm. **l**, Quantification of LC axon density and microglia density in the anterior Pir at 3 months of age. **m**, Quantification of LC axon density and microglia density in the anterior Pir at 6 months of age. **n**, Aβ plaque load in the anterior Pir of $APP^{NL\text{-}G\text{-}F}$ animals at 3 and 6 months of age. **o**, Immunostaining against LC axons (NET, magenta), microglia (Iba1, green) and Aβ plaques (Aβ, red) in the posterior piriform cortex. Scale bar: 50 µm. **p**, Quantification of LC axon density and microglia density in the posterior Pir at 3 months of age. **q**, Quantification of LC axon density and microglia density in the posterior Pir at 6 months of age. **r**, Aβ plaque load in the posterior Pir of $APP^{NL\text{-}G\text{-}F}$ animals at 3 and 6 months of age.; Data expressed as mean ± s.e.m.; ns, not significant; **p<0.01. Statistics shown in Supplementary Data 2. Source data are provided as a Source Data file.

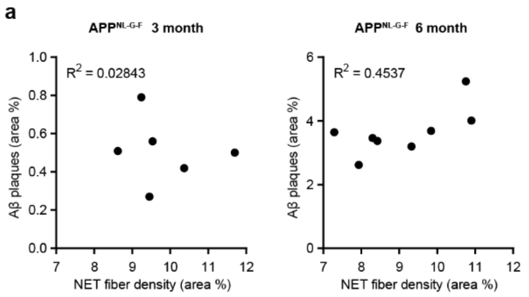

Supplementary Fig. 2: LC axon loss is independent of extracellular Aβ plaques

a, Correlation between NET fibre density and Aβ plaque load in the OB of $APP^{NL\text{-}G\text{-}F}$ mice at 3 and 6 months of age; Data expressed as mean. Statistics shown in Supplementary Data 2. Source data are provided as a Source Data file.

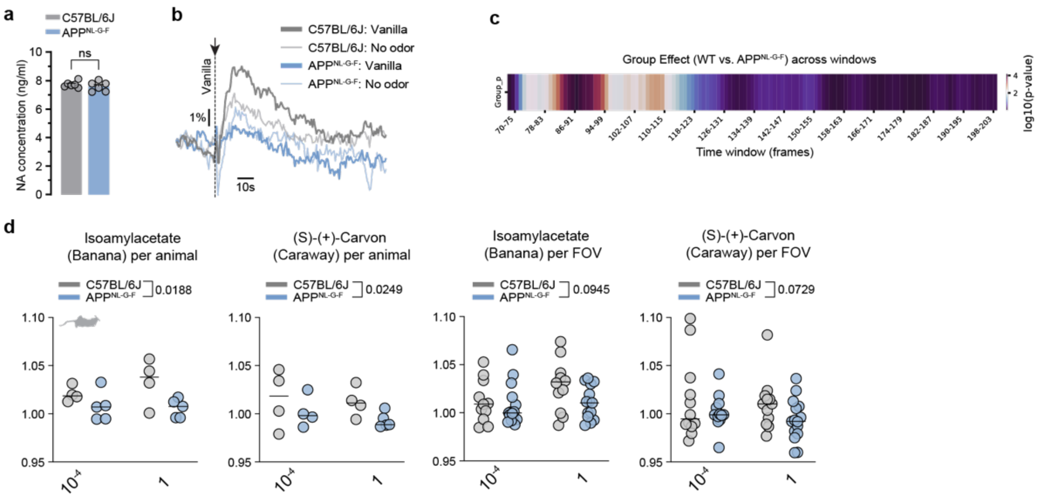

Supplementary Fig. 3: Noradrenaline measurements

a, Noradrenaline (NA) concentration in the olfactory bulb assessed by NA ELISA shows no difference between the groups in 3 months old animals. **b**, NA response to a vanilla air puff or a control air puff compared between C57BL/6J and $APP^{NL\text{-}G\text{-}F}$ mice. **c,** Heatmap with p-values frame-by-frame across tested odours identifying the analysis window (frame 86-91). **d,** In vivo 2P imaging of NA with to different odour dilutions (undiluted and 1:1000). Data expressed as mean ± s.e.m.; ns, not significant. Statistics shown in Supplementary Data 2. Source data are provided as a Source Data file.

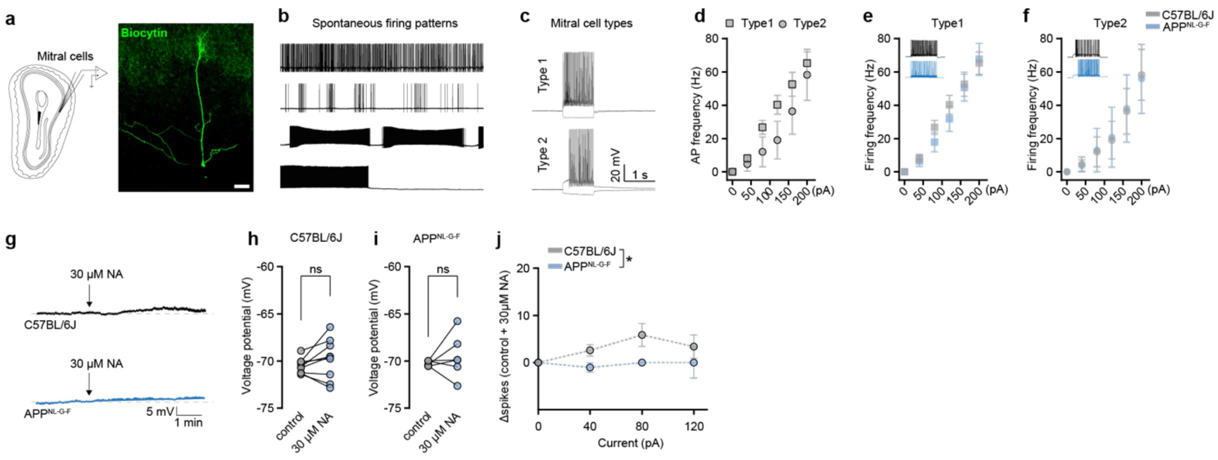

Supplementary Fig. 4: Electrophysiological properties of mitral cells in the olfactory bulb

a, Perforated patch-clamp recordings with subsequent biocytin-filling and post-hoc staining (green) visualizes a mitral cell in the OB. Scale bar: 20 µm. **b**, Diverse spontaneous firing patterns of mitral cells. **c**, Two different mitral cell-types were identified based on the cell's response to current stimulations. **d**, Current-frequency curve comparing type 1 and type 2 mitral cells in C57BL/6J animals. **e**, Current-frequency curve of type 1 mitral cells and **f**, type 2 mitral cells in C57BL/6J and $APP^{NL\text{-}G\text{-}F}$ mice. **g**, Voltage potential difference before (control) and after 30 µM noradrenaline (NA) bath application. **h and i**, Voltage potential difference before (control) and after 30 µM noradrenaline bath application in C57BL/6J and $APP^{NL\text{-}G\text{-}F}$ animals at 6 months of age. **j** Current-frequency curve comparing C57BL/6J and $APP^{NL\text{-}G\text{-}F}$ mitral cell firing behaviour in response to noradrenaline application; Data expressed as mean ± s.e.m.; ns, not significant. Statistics shown in Supplementary Data 2. Source data are provided as a Source Data file.

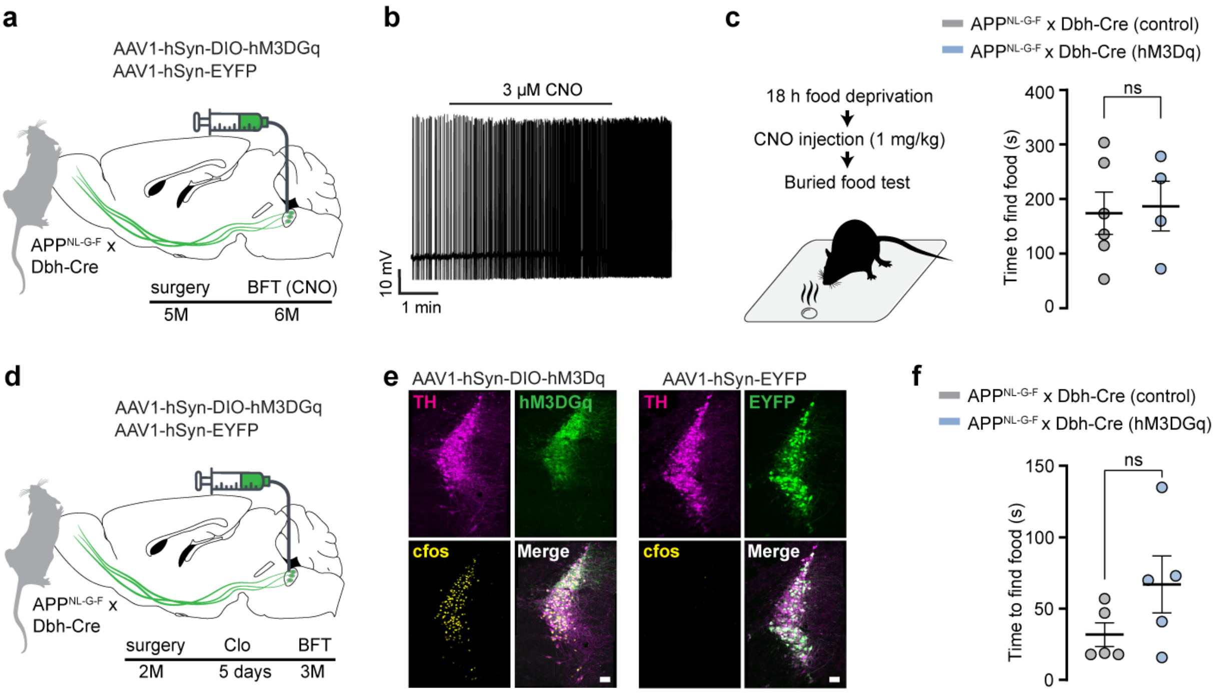

Supplementary Fig. 5: Chemogenetic over-activation of LC neurons cannot reinstate olfaction

a, Experimental setup of DREADD-virus injection into the LC of APP^{NL-G-F} x Dbh-Cre mice at 5 months of age. **b**, Electrophysiological recording of the spontaneous action potential firing of an LC neuron. Bath application of CNO increases the spontaneous firing frequency. **c**, Buried foot test after CNO injection showed no change in the time to find a buried food pellet. **d**, Experimental setup of DREADD-virus injection into the LC of APP^{NL-G-F} x Dbh-Cre mice at 2 months of age and Clozapine (Clo) application for 5 consecutive days before the BFT at 3 months of age. **e**, Immunostaining visualizing neuronal activity (c-fos, yellow) of LC neurons (TH, magenta) after hM3DGq (green) activation with Clo. **f**, Buried foot test after 5 days of Clo injection showed no difference in the time to find a buried food pellet; Data expressed as mean ± s.e.m.; ns, not significant; Statistics shown in Supplementary Data 2. Source data are provided as a Source Data file.

a

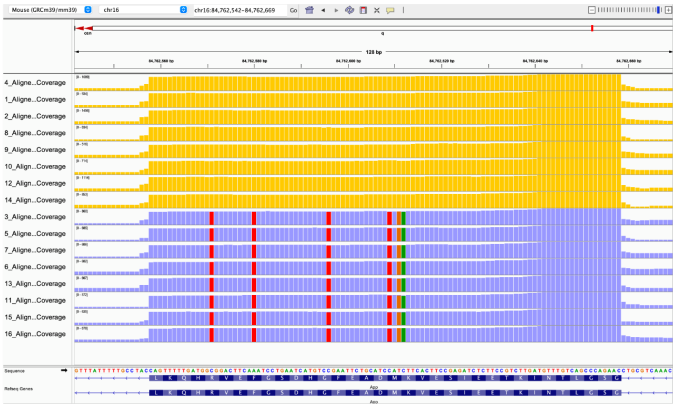

b

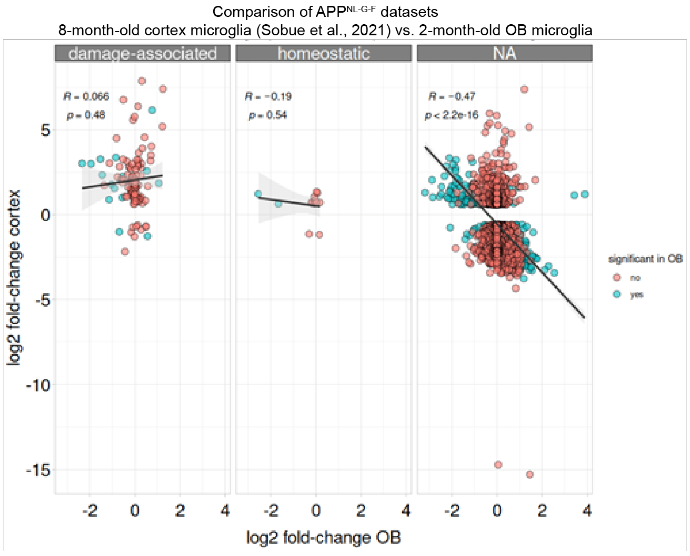

Supplementary Fig. 6: RNA sequencing control

a, IGV browser screenshot showing RNA-Seq alignments to the APP gene (GRCm39) and confirming the $APP^{NL\text{-}G\text{-}F}$ transgene presence in transgene animals (blue bars) compared to C57BL/6J animals (yellow bars). Red bars in the sequence demonstrate C->T or A->T mutations, while ochre bars indicate T->C and green bars C->A mutations. **b,** Linear regression of log-fold changes reveal a significant negative relationship (R = -0.44, p < 2e-16) between young OBs and aged cortex. Transcriptomic data can be found at Sequence Read Archive (SRA) under accession code SRP596060.

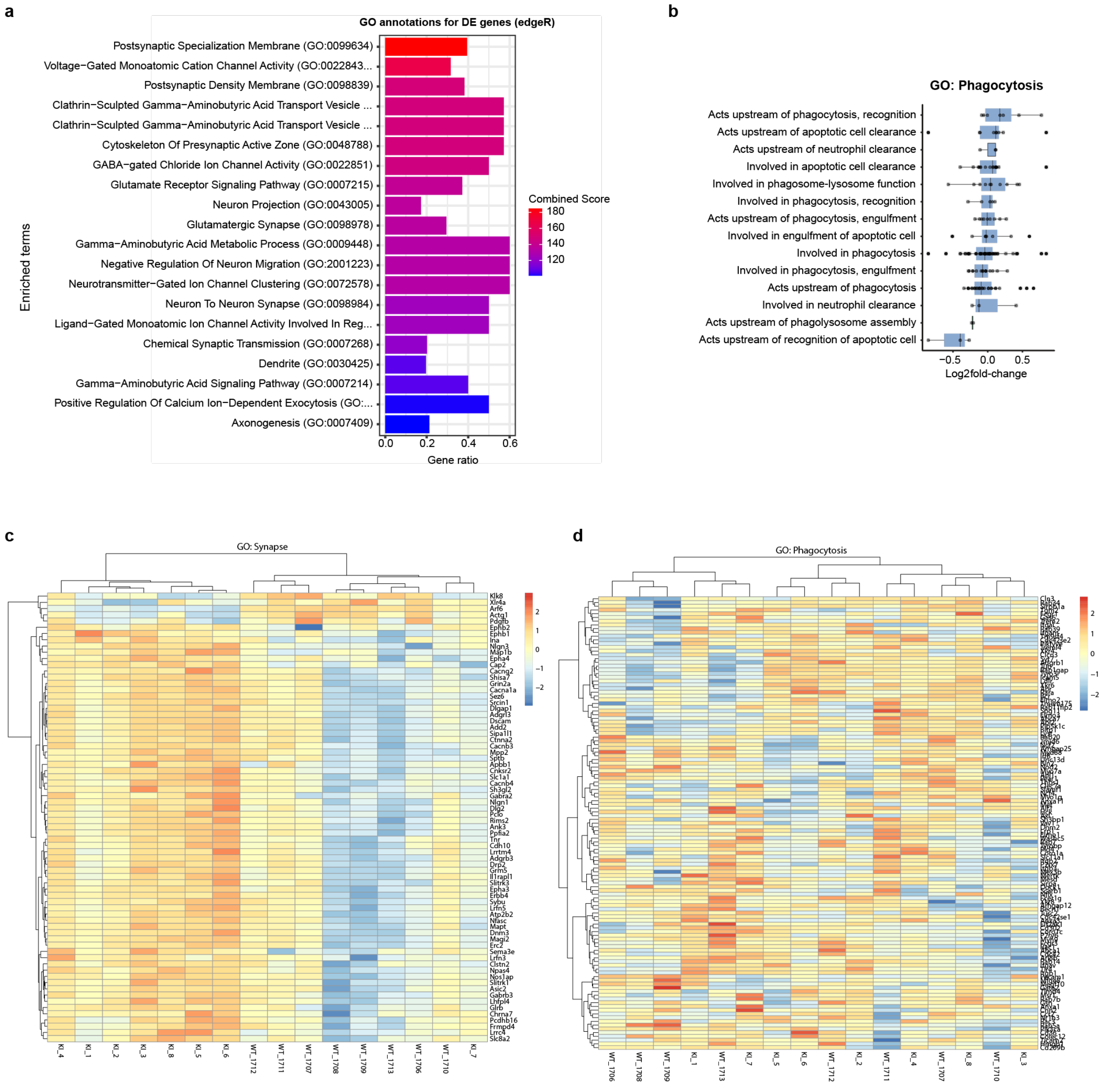

Supplementary Fig. 7: Gene ontology annotations

a, 20 most enriched gene ontology (GO) annotations for differentially upregulated genes. **b**, GO enrichment analysis of genes involved in phagocytosis. **c,** Heatmap illustrating DE of genes annotated with “Synapse” and “Phagocytosis” **(d)**. Transcriptomic data can be found at GEO under ID GSE302245 (https://www.ncbi.nlm.nih.gov/geo/query/acc.cgi?acc=GSE302245).

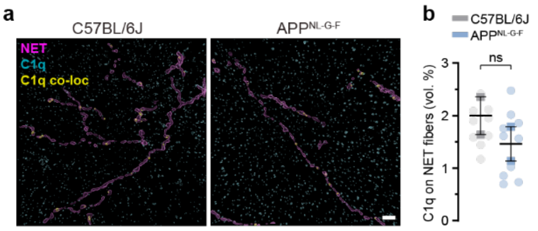

Supplementary Fig. 8: C1q does not serve as "eat-me" signal on LC axons

a, 3D reconstruction of LC axons (NET, magenta), C1q (cyan) and C1q signals colocalised to LC axons (yellow). Scale bar: 5 µm. **b**, Quantification of C1q signal colocalised to LC axons; Data expressed as mean ± s.e.m.; ns, not significant. Statistics shown in Supplementary Data 2. Source data are provided as a Source Data file.

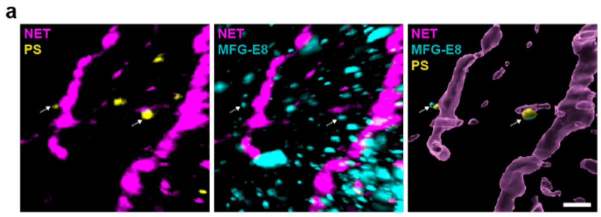

Supplementary Fig. 9: LC axons are tagged with phosphatidylserine (PS) and MFG-E8

a, Immunostaining and 3D reconstruction of PS (yellow) and MFG-E8 (cyan), colocalised to LC axons (NET, magenta). Scale bar: 2 µm.

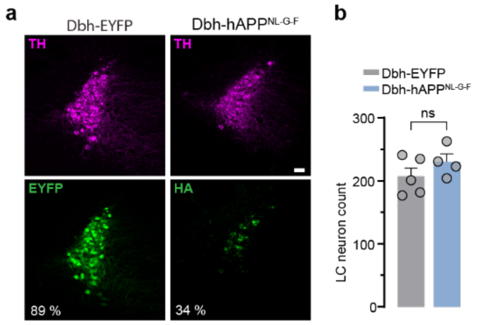

Supplementary Fig. 10: Expression of APP^{NL-G-F} virus in the LC

a, Immunostaining of LC neurons (TH, magenta) and the respective fluorophore expression of each virus (Dbh-EYFP: EYFP and Dbh-hAPP^{NL-G-F}: HA-tag). Scale bar: 50 µm **b**, Neuron

count shows no difference in LC neuron number; Data expressed as mean ± s.e.m.; ns, not significant. Statistics shown in Supplementary Data 2. Source data are provided as a Source Data file.

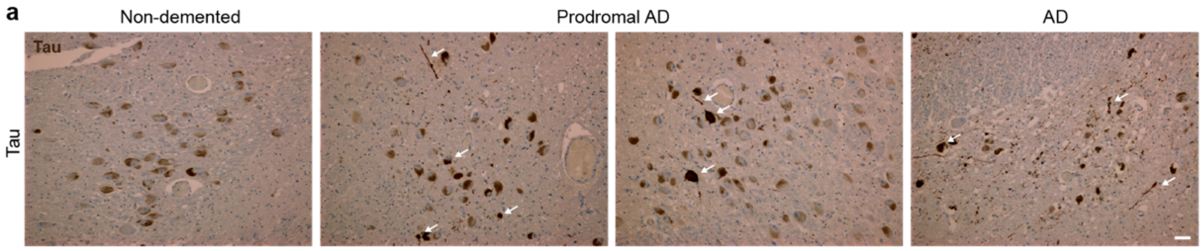

Supplementary Fig. 11: Tau-pathology in LC of brain donors

a, Immunohistochemical staining of human locus coeruleus brain sections stained against Tau (Tau, brown). In the non-demented control, healthy neuromelanin-containing LC neurons have a light brown appearance. Arrows in the prodromal and AD pictures point at dark-brown neurofibrillary tangle. Scale bar: 50 µm.

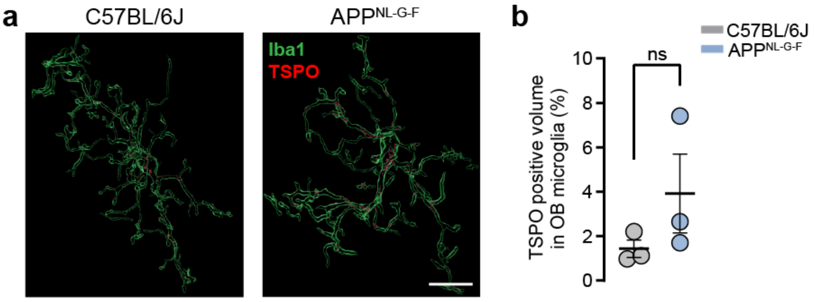

Supplementary Fig. 12: TSPO expression in microglia of OB from APP$^{NL\text{-}G\text{-}F}$ mice

a, Representative images of TSPO (TSPO, red) reconstruction in microglia (Iba1, green). Scale bar: 20 µm. **b,** TSPO volume relative to Iba1^{+} volume in OB sections from WT and APP$^{NL\text{-}G\text{-}F}$ mice at the age of 3 months. Each point represents the mean for a single animal. Data expressed as mean ± s.e.m.; ns, not significant. Statistics shown in Supplementary Data 2. Source data are provided as a Source Data file.
