# Supplementary material for: Early Locus Coeruleus noradrenergic axon loss drives olfactory dysfunction in Alzheimer’s disease
Source: Nat Commun. 2025 Aug 8;16:7338. doi: 10.1038/s41467-025-62500-8 (PMC12334674; doi:10.1038/s41467-025-62500-8)
Supplement: Supplementary file 2 — Description of Additional Supplementary Files [file 41467_2025_62500_MOESM2_ESM.pdf]

Description of Additional Supplementary Files

Supplementary Data 1: Shown are the statistics used in main figures 1-6. In detail, the comparing groups, statistical tests and related measures, number of animals and number of replicates are listed.

Supplementary Data 2: Shown are the statistics used in supplementary figures 1-12. In detail, the comparing groups, statistical tests and related measures, number of animals and number of replicates are listed.

Supplementary Data 3: The data provide an overview about the demographic details of human participants. Supplementary Data from Fig. 6a-c shows the demographic details of subjects donating brain tissue. The age, gender and neuropathological diagnosis is listed for every subject. Based on immunohistological stains, a Thal-phase and Braak stage was assigned to every subject, as well as the co-pathology of Tau and α-synuclein in the locus coeruleus assessed. Supplementary Data from Fig. 6d-f shows the demographic details of subjects participating in the TSPO-PET and olfaction study. The age, gender and cognitive diagnosis is listed for every subject. Additionally, Aβ-levels and Tau-levels, measured in the cerebrospinal fluid, are listed. TSPO-PET signals measured in the OB are given as the concentration of the radioactive tracer in the tissue (SUVr, Standardized Uptake Value Ratio). The evaluation of the olfaction is defined as the percentage of correctly identified sticks.
