# Supplementary material for: Early Locus Coeruleus noradrenergic axon loss drives olfactory dysfunction in Alzheimer’s disease
Source: Nat Commun. 2025 Aug 8;16:7338. doi: 10.1038/s41467-025-62500-8 (PMC12334674; doi:10.1038/s41467-025-62500-8)
Supplement: Supplementary file 6 — Reporting Summary [file 41467_2025_62500_MOESM6_ESM.pdf]

nature portfolio

Corresponding author(s): Lars Paeger

Last updated by author(s): Jul 11, 2025

Reporting Summary

Nature Portfolio wishes to improve the reproducibility of the work that we publish. This form provides structure for consistency and transparency in reporting. For further information on Nature Portfolio policies, see our Editorial Policies and the Editorial Policy Checklist.

Statistics

For all statistical analyses, confirm that the following items are present in the figure legend, table legend, main text, or Methods section.

n/a	Confirmed	
☐	☒	The exact sample size (*n*) for each experimental group/condition, given as a discrete number and unit of measurement
☐	☒	A statement on whether measurements were taken from distinct samples or whether the same sample was measured repeatedly
☐	☒	The statistical test(s) used AND whether they are one- or two-sided<br>*Only common tests should be described solely by name; describe more complex techniques in the Methods section.*
☒	☐	A description of all covariates tested
☐	☒	A description of any assumptions or corrections, such as tests of normality and adjustment for multiple comparisons
☐	☒	A full description of the statistical parameters including central tendency (e.g. means) or other basic estimates (e.g. regression coefficient) AND variation (e.g. standard deviation) or associated estimates of uncertainty (e.g. confidence intervals)
☐	☒	For null hypothesis testing, the test statistic (e.g. *F*, *t*, *r*) with confidence intervals, effect sizes, degrees of freedom and *P* value noted<br>*Give P values as exact values whenever suitable.*
☐	☒	For Bayesian analysis, information on the choice of priors and Markov chain Monte Carlo settings
☐	☒	For hierarchical and complex designs, identification of the appropriate level for tests and full reporting of outcomes
☐	☒	Estimates of effect sizes (e.g. Cohen's *d*, Pearson's *r*), indicating how they were calculated

Our web collection on statistics for biologists contains articles on many of the points above.

Software and code

Policy information about availability of computer code

Data collection	ZEN v3.1 (Zeiss); Incucyte™ S3 Live-Cell Analysis System (Sartorius); PatchMaster v2x90.5; Arch Linux, R version 4.3.2; Rstudio Server 2023.03.0; NextSeq 550 sequencer (Illumina, 20024907); MES v8.3.1458 (Femtonics)
Data analysis	Fiji v1.54f; GraphPad Prism 10; IMARIS v9.6.1; Incucyte GUI v2021A; Spike2 v10.13; Matlab R2022b; Python; S.L.E.A.P. networks (PMID: 35379947); PMOD (V3.5 PMOD Technologies)

For manuscripts utilizing custom algorithms or software that are central to the research but not yet described in published literature, software must be made available to editors and reviewers. We strongly encourage code deposition in a community repository (e.g. GitHub). See the Nature Portfolio guidelines for submitting code & software for further information.

Data

Policy information about availability of data

All manuscripts must include a data availability statement. This statement should provide the following information, where applicable:

- Accession codes, unique identifiers, or web links for publicly available datasets
- A description of any restrictions on data availability
- For clinical datasets or third party data, please ensure that the statement adheres to our policy

Source data are provided for figures: 1c/d/f/g/i/k/m/n; 2f/g/i; 3b/i/k; 4b/c/e/g/i/k/n/o/q; 5c-e/g; 6b/c/e/f/h/i/j and supplementary figures: 1b/c/d/f/g/i/j/l/m/n/p/q/r; 2a; 3a/b; 4h/i/j; 5c/f; 8b; 10b, 12b.RNA-sequencing data are provided at https://www.ncbi.nlm.nih.gov/geo/query/acc.cgi?acc=GSE302245. RNA-sequencing data from 8 month old APP-NL-G-F mice (Sobue et al., 2021) were publicly available (https://actaneurocomms.biomedcentral.com/articles/10.1186/s40478-020-01099-

x#Sec21, additional file 1). Demographic details of human subjects can be found in Supplementary Data 3. Further information are available on request to the corresponding author.

Research involving human participants, their data, or biological material

Policy information about studies with human participants or human data. See also policy information about sex, gender (identity/presentation), and sexual orientation and race, ethnicity and racism.

Reporting on sex and gender	Male and female human post-mortem tissue was analyzed. The sex was balanced between the groups and no difference between sex was observed; Participants taking part in the TSPO-PET and Sniffing stick test study had both sex and no difference between the sex was observed.
Reporting on race, ethnicity, or other socially relevant groupings	Patients donating brain tissue and patients participating in the TSPO-PET / Sniffing stick study are of Caucasian ethnicity.
Population characteristics	Demographic details of human subjects can be found in the Supplementary Data 3.
Recruitment	Human post-mortem tissue was provided by the Munich brain bank. Prior to death, patients volunteered to donate tissue by agreeing to the information sheet provided by the brain bank.<br>Participants from the TSPO-PET and Sniffing stick study were recruited through the Alzheimer Therapy and Research Center at the Department of Psychiatry, LMU University Hospital Munich.
Ethics oversight	The use of human post-mortem tissue was approved by the Ethics Committee of the Ludwig-Maximilians University Munich. The human TSPO-PET / Sniffing stick study was approved by the Ethics Committee of LMU University Hospital Munich.

Note that full information on the approval of the study protocol must also be provided in the manuscript.

Field-specific reporting

Please select the one below that is the best fit for your research. If you are not sure, read the appropriate sections before making your selection.

☒ Life sciences ☐ Behavioural & social sciences ☐ Ecological, evolutionary & environmental sciences

For a reference copy of the document with all sections, see nature.com/documents/nr-reporting-summary-flat.pdf

Life sciences study design

All studies must disclose on these points even when the disclosure is negative.

Sample size	Both for in vitro and in vivo experiments, the number of animals was determined empirically, based on previous experiments with the used mouse lines, publications and technical feasibility. At least 3 animals per group were used to conduct basic statistical analysis. All sample sizes are listed in Supplementary Data 1.
Data exclusions	Statistical outliers were identified and removed using the Grubbs' test (for a single outlier) or the ROUD method (for several outliers) in the software PRISM 10. Virus injected animals were excluded if the virus expression was not successful.
Replication	Immunostainings and the olfactory behavioral tests on mice were conducted with different animal batches to replicate data. Immunostainings were in addition run with 3-6 technical replicates. For RNA sequencing and the phagocytosis assay 3 technical replicates were used. Virus injections and subsequent behavioral tasks were only performed once and used animals were from the same batch to ensure that all animals were of the same age.
Randomization	For virus injections into animals of the same genotype, animals were randomly assigned to the treatment groups. For all other experiments, randomization was not possible because groups were predefined by genotype.
Blinding	Most experiments could not be performed in a blinded manner as mice and samples needed to be assigned to their identification number to match them to the experimental groups. The analysis of the buried food test was performed in a blinded manner.

Reporting for specific materials, systems and methods

We require information from authors about some types of materials, experimental systems and methods used in many studies. Here, indicate whether each material, system or method listed is relevant to your study. If you are not sure if a list item applies to your research, read the appropriate section before selecting a response.

Materials & experimental systems

n/a	Involved in the study
[ ]	[x] Antibodies
[x]	[ ] Eukaryotic cell lines
[x]	[ ] Palaeontology and archaeology
[ ]	[x] Animals and other organisms
[x]	[ ] Clinical data
[x]	[ ] Dual use research of concern
[x]	[ ] Plants

Methods

n/a	Involved in the study
[x]	[ ] ChIP-seq
[x]	[ ] Flow cytometry
[x]	[ ] MRI-based neuroimaging

Antibodies

Antibodies used

rabbit anti-NET (1:500, Abcam, ab254361), mouse anti-NET (1:1000, Thermo Fisher, MA5-24547), guinea pig anti-Iba1 (1:500, Synaptic Systems, 234308), chicken anti-TH (1:1000, Abcam, ab76442), mouse anti-Aß (NAB228) (1:500, Santa Cruz, sc-3277), rat anti-CD68 (1:500, BioRad, MCA1957), goat anti-MFG-E8 (1:500, R&D Systems, AF2805), rabbit anti-C1q (1:1000, Abcam, ab182451), chicken anti-GFP (1:1000, Abcam, ab13970), rabbit anti-GFP (1:1000, Thermo Fisher, A21311), rabbit, HA-tag (1:500, Sigma, H6908), Streptavidin 488 (1:1000, Invitrogen, S32354), Streptavidin 647 (1:1000, Invitrogen, S32357).

Validation

All antibodies used were commercially available and selected based on publications and the manufacturer's validation. A secondary antibody control was used to validate the specificity of the stain.

Animals and other research organisms

Policy information about studies involving animals; ARRIVE guidelines recommended for reporting animal research, and Sex and Gender in Research

Laboratory animals

Mice, 1-6 months of age, of the following lines were used: C57BL/6J, Dbh-Cre, APP-NL-G-F (Apptm3.1Tcs/Apptm3.1Tcs), APP-NL-G-F x TSPO-KO and APP-NL-G-F x Dbh-Cre.

Wild animals

No wild animals were used in this study.

Reporting on sex

Male and female mice were used in this study. No differences based on sex were found in the following experiments: Immunohistological stainings, electrophysiology, and phagocytosis assay. In the buried food test, at 6 month of age, males of both genotypes performed worst than females. RNA sequencing data showed female mice to have more differentially expressed genes than males. For the in vivo noradrenaline measurement and the AVV-APP virus injections only males were used. Small animal PET imaging was performed on female mice only due to housing and farewell reasons.

Field-collected samples

No field-collected samples were used in this study.

Ethics oversight

The facility and all animal experiments are in accordance with official animal protection standards and reviewed and approved by an Institutional Animal Care and Use Committee and the office for public health and consumer protection of Upper Bavaria's government. The specific and opportunistic pathogen-free (SOPF)-mouse facilities are controlled and registered by the Department of Public Order (KVR-I/221) with approved animal welfare licenses.

Note that full information on the approval of the study protocol must also be provided in the manuscript.

Plants

Seed stocks

n/a

Novel plant genotypes

n/a

Authentication

n/a
